# Time-Dependent Modulation of Phenolics, Polysaccharides, and Bioactivities in *Artemisia argyi* Leaves During Solid-State Fermentation with *Rhizopus oryzae*

**DOI:** 10.3390/foods14244262

**Published:** 2025-12-11

**Authors:** Hongzhi Chen, Xiuren Zhou, Lei Dai

**Affiliations:** 1 Department of Bioengineering, Xinxiang Institute of Engineering, 777# Xinfei Avenue South Section, Xinxiang 453700, China; chenhongzhi@xxgc.edu.cn; 2 School of Life Sciences, Henan Institute of Science and Technology, Huanlan Road, 655#, Xinxiang 453002, China; daileinihao@126.com

**Keywords:** *Artemisia argyi*, solid-state fermentation, *Rhizopus oryzae*, biotransformation, antioxidant activity, antidiabetic activity, secondary metabolites, plant foods

## Abstract

This study investigated the biotransformation of bioactive compounds in *Artemisia argyi* leaves, a traditional plant food, via solid-state fermentation (SSF) with *Rhizopus oryzae*. Over a 10-day period, total phenolic and flavonoid contents increased by 241.46% and 104.67%, respectively, while polysaccharides peaked on day 2. Antioxidant activity was significantly enhanced, with the values of half maximal inhibitory concentration (IC50) for DPPH, ABTS, hydroxyl, and superoxide anion radicals reduced by up to 59.26%. Chemical characterization via GC-MS revealed selective enrichment of oxygenated monoterpenes (e.g., eucalyptol, borneol), indicating fungal-mediated conversion of secondary metabolites. Fermented extracts exhibited potent xanthine oxidase inhibition (34.04% at 100 μg/mL), indicating potential anti-hyperuricemic activity, and tyrosinase inhibition (50.02%). Compared to liquid-state fermentation (LSF), previous studies have reported that SSF by *R. oryzae* provides a sustainable, low-moisture fermentation mode that can enhance metabolite stability and help preserve volatile components. These findings highlight an effective biotransformation strategy for valorizing herbal materials with enhanced antioxidant and enzyme inhibitory properties.

## 1. Introduction

Biological fermentation has become one of the key technological tools for enhancing the active components and functional properties of plant materials, including herbs and health supplements [1,2,3], or animal-derived ones, including milk and meat [4,5,6]. Throughout human history, fermentation has frequently been employed in food production. For instance, in East Asia, soybeans have long been used to produce foods like fermented black beans and soy sauce through fermentation by various microorganisms. In Western countries, the production of yogurt and cheese also relies on microbial fermentation processes [7,8,9,10]. This demonstrates that carefully controlled microbial activity can enhance nutritional quality and sensory attributes. Currently, fermentation processes are not only widely applied in the production of traditional fermented foods but also play a crucial role in manufacturing various active pharmaceutical ingredients. Microorganisms are known to synthesize various metabolites such as organic acids, enzymes, antibiotics, and vitamins [11,12,13,14]. When applied to plant materials, these microbial activities can modify the phytochemical composition by releasing bound compounds or generating new bioactive molecules [15,16]. This relevance provides the foundation for exploring how solid-state fermentation (SSF) may enhance the functional properties of herbal materials such as *Artemisia argyi*.

Although liquid-state fermentation (LSF) is presently the common method for enhancing active substances and antioxidant compounds in medicinal plants, SSF offers a green, low-carbon alternative [17,18]. Compared to the former, SSF typically exhibits more efficient resource utilization and can mimic the natural growth of microorganisms on solid substrates while requiring minimal water and energy consumption [17,19,20]. Additionally, SSF reduces environmental pollution risks and increases the final concentration of target products. This makes it a highly promising option for sustainably enhancing the bioactive components and physiological activity of medicinal herbs [21,22]. When herbal residues such as *Salvia miltiorrhiza* undergo SSF by fungi like *Aspergillus oryzae*, both phenolic levels and biological activity show significant increases [23,24]. Similarly, after fungal SSF, the free radical scavenging and hypoglycemic effects of *Smilax glabra* are markedly improved [25].

However, whether similar SSF-induced enhancements occur in *A. argyi* leaves and how these changes progress over the course of fermentation remain unclear. *A. argyi* is a perennial herb widely recognized in traditional pharmacopeias. It is highly valued for its pronounced anti-inflammatory, antibacterial, and circulation-promoting properties and is commonly used to treat respiratory and gynecological conditions [15,26,27]. *A. argyi* leaves have been found to contain volatile compounds and phenolic substances, and animal studies have demonstrated their ability to regulate immune responses and improve gut health [28,29]. *A. argyi* leaves are rich in phenolic compounds, predominantly flavones and phenolic acids such as eupatilin, jaceosidin, apigenin, luteolin, and chlorogenic acid, providing a biochemical basis for examining changes in total phenolics and flavonoids during SSF [30,31]. Fermentation has been increasingly explored as an approach to improving the quality and bioactivity of *A. argyi*. Previous studies using LSF with lactic acid bacteria, Lactiplantibacillus, or Monascus species have demonstrated modifications in phenolic composition, enhancements in antioxidant capacity, and alterations in the volatile profile [32,33,34]. Microbial or enzymatic treatments have also been applied to mimic some of the biochemical transformations that occur during traditional long-term aging [35,36]. However, despite these advances, little is known about how bioactive components of *A. argyi* change during SSF, particularly when fermented with *Rhizopus oryzae*. Although *R. oryzae* is generally known to produce hydrolytic enzymes capable of degrading polysaccharides and releasing bound phenolics in various plant substrates [37,38], direct evidence demonstrating these effects specifically in *A. argyi* is limited. Furthermore, no systematic study has characterized time-resolved changes in phenolics, polysaccharides, enzyme-inhibitory activities, and volatile metabolites during SSF of *A. argyi*.

In this research, we hypothesized that SSF with *R. oryzae* would promote the release or transformation of bioactive constituents through enzymatic hydrolysis and that a 10-day SSF process could partially mimic certain biochemical features typically observed after extended natural aging. Based on this hypothesis, we conducted a comprehensive, time-resolved evaluation of physicochemical properties, active ingredient contents, enzyme inhibition activities, and volatile profiles of *A. argyi* leaves during SSF, aiming to enhance the herb’s therapeutic efficacy by increasing its bioactivity via an environmentally friendly process.

## 2. Materials and Methods

### 2.1. Sample Collection and Fermentation Substrate Preparation

Fresh leaves of *A. argyi* were collected in early June 2024 from cultivated plants growing in Sanhe Dian Village, Huixian City, Henan Province, China. The plants were harvested at the vigorous vegetative stage, shortly before flowering. Species identity was authenticated by Dr. Pengming Yang (Henan Institute of Science and Technology, Xinxiang, China). A voucher specimen set was deposited in the Herbarium of Henan Institute of Science and Technology under voucher numbers Hp2405001–Hp2405010. The material was air-dried initially, followed by forced-air drying at 70 °C until moisture was below 5%. The dried leaves were ground to a uniform particle size of approximately 1 mm using a laboratory mill. For the fermentation medium, glucose (1% *w*/*w*) and peptone (0.2% *w*/*w*) were added as the carbon and nitrogen sources, respectively. The supplementation of glucose and peptone was selected based on commonly used formulations in SSF systems reported in the literature and preliminary optimization in our laboratory to ensure sufficient carbon and nitrogen availability for *R. oryzae* growth. For solid-state fermentation, fresh *A. argyi* leaves were weighed after moistening, and 1000 g (wet weight, after adding 500 mL of sterile water) of leaf material was loaded into each 3000 mL Erlenmeyer flask (borosilicate glass). The sterilized flasks provided an internal geometry suitable for uniform substrate spreading, and the leaf substrate formed a layer approximately 13–15 cm in height. Prior to inoculation, the moistened substrate exhibited an initial pH of 7.2, measured using a calibrated pH meter. All fermentation vessels were sterilized at 121 °C for 30 min before use.

Aged *A. argyi* leaves were prepared following the conventional three-year natural aging method widely used in traditional processing. Briefly, the leaves used for the aged control were first air-dried in a well-ventilated, shaded environment for approximately 10–14 days until constant weight before being stored for natural aging. Then, placed in double-layer kraft paper bags, and stored indoors at ambient room temperature (18–28 °C) under well-ventilated conditions with approximate relative humidity of 45–70%. The leaves were protected from direct sunlight and turned periodically to prevent moisture accumulation. After three years of storage, the aged samples were collected and used as the aged control group in this study.

Fresh leaves for solid-state fermentation are dried in an oven at 70 °C, a temperature commonly used in *A. argyi* laboratory processing. This drying temperature enables rapid moisture removal, reduces enzymatic browning, and minimizes microbial contamination. While temperatures above 60 °C may affect certain heat-sensitive phenolic and volatile compounds, 70 °C represents a widely accepted compromise that preserves key chemical constituents while ensuring safe and consistent sample preparation. In contrast, aged control leaves were processed using a traditional three-year natural aging method, wherein leaves were air-dried at ambient temperatures and subsequently stored indoors under ventilated conditions. These differing drying approaches reflect their respective processing objectives; thus, observed variations between fermented and aged samples represent both fermentation-induced changes and characteristics inherent to natural aging.

It is important to note that only the substrates used for solid-state fermentation underwent autoclave sterilization (121 °C, 30 min). The fresh-dried leaves (dried at 70 °C) and the three-year aged leaves (air-dried naturally) were not subjected to sterilization to preserve their traditional processing characteristics. Therefore, differences between fermented samples and the two non-fermented controls may partially include effects of the sterilization step, in addition to the biochemical changes caused by fermentation.

Fresh dried leaves refer to *A. argyi* leaves that were air-dried in a shaded, well-ventilated environment at ambient temperature for 10–14 days until constant weight, without any subsequent aging. Aged leaves refer to leaves that were first air-dried under the same shaded conditions and then stored under natural ventilation for three years to obtain the traditionally aged material.

### 2.2. Inoculum Development for R. oryzae

*R. oryzae* strain ATCC 34612 was cultured on slant agar media containing potato extract and dextrose at 28 °C for 96 h to promote sporulation. The inoculum of *R. oryzae* strain ATCC 34612 was prepared by washing spores from 96 h culture grown on potato dextrose agar (PDA) at 30 °C. The resulting spore suspension was adjusted to approximately 1 × 10^7^ spores/mL. Colony-forming units (CFUs) were estimated using serial dilution and plating on PDA, followed by counting colonies after incubation at 30 °C for 48 h. For solid-state fermentation, the moistened *A. argyi* substrate was inoculated at 5% (*v*/*w*), corresponding to 50 mL of spore suspension per 1000 g of wet substrate. The inoculum was evenly distributed by manual mixing to ensure uniform fungal colonization. The inoculated material was maintained at 30 °C with 75–85% humidity in a controlled chamber for 10 days, with manual turning every 48 h to ensure uniform growth and oxygenation.

Each fermentation time point (0, 2, 4, 6, 8, and 10 days) was obtained from independent fermentation vessels, each inoculated separately under identical conditions. For every time point, three individual Erlenmeyer flasks were prepared as biological replicates, and no vessel was sampled more than once. This design ensured independence among replicates and allowed valid statistical comparisons using one-way ANOVA.

In this study, the 0-day fermentation sample refers to the substrate after moisture adjustment and autoclave sterilization but immediately after inoculation, before any visible mycelial growth occurred. This sample thus represents the initial physicochemical state of the sterilized substrate at the start of fermentation (day 0).

### 2.3. Extraction of Bioactive Compounds

For phenolic and flavonoid extraction, 1 g of powdered sample (fermented or control) was suspended in 20 mL of 70% methanol, vortexed, and sonicated at 40 kHz for 45 min at ambient temperature (approximately 25 °C). Each extraction was conducted once per sample, and the solvent volume and sample mass were maintained consistently across all treatments to ensure comparability. The supernatant was recovered after centrifugation (10,000× *g*, 15 min) and used for analyses. Polysaccharides were isolated by hot water extraction: a 5 g sample in 100 mL distilled water at 90 °C for 2 h, followed by precipitation with 95% ethanol (1:4 *v*/*v*) overnight at 4 °C, and redissolution in water. For essential oil extraction, 100 g of *A. argyi* leaf material was placed into a Clevenger-type apparatus together with 1000 mL of distilled water. Hydrodistillation was performed for 3 h, and the distillation time was recorded starting from the appearance of the first drop of distillate in the condenser. The essential oils obtained by hydrodistillation were immediately dried over anhydrous sodium sulfate, transferred into amber glass vials, and stored at −20 °C in darkness. All samples were analyzed by GC-MS within two weeks of extraction to minimize potential storage-related changes in volatile composition.

### 2.4. Quantification of Active Ingredients

Total flavonoids were determined colorimetrically using NaNO_2_-Al(NO_3_)_3_ complex formation, measured at 510 nm, and expressed as rutin equivalents [39]. The total phenolic content was determined using gallic acid as a standard through the phosphomolybdic phosphotungstic acid assay, with absorbance measured at 765 nm [40]. Polysaccharide content was quantified using the anthrone sulfuric acid method at 620 nm, employing glucose as the standard [41].

All quantitative measurements of total phenolics, total flavonoids, and polysaccharides were expressed on a dry weight basis (mg/g·DW). For each assay, the concentrations obtained from calibration curves were converted to mg equivalents per gram of dried *A. argyi* leaf material. This dry weight normalization was applied consistently across all treatments, including fresh dried, fermented, and aged samples, and the same units (mg/g·DW) are reported in Table 1 to ensure methodological consistency and comparability.

### 2.5. Assessment of Antioxidant Capacity

Free radical scavenging was evaluated using multiple systems: DPPH (517 nm, ascorbic acid standard) [42], ABTS (734 nm, Trolox standard) [43], hydroxyl radical (Fenton reaction, 510 nm) [44], and superoxide anion (NBT reduction, 560 nm) [45]. The values of half maximal inhibitory concentration (IC50) were calculated from dose response curves (10–600 μg/mL sample concentrations) via nonlinear regression [46]. Free radical scavenging assays were conducted using the methanolic extracts directly obtained after filtration. For each assay, the stock extracts were diluted with methanol to final concentrations ranging from 10 to 600 μg/mL. All dilutions were prepared freshly prior to measurement to ensure stability of the radical-scavenging activity.

Antioxidant activities were evaluated using assays following standardized procedures with minor modifications. For the DPPH assay, 100 µL of sample extract was mixed with 3.9 mL of 0.1 mM DPPH solution prepared in methanol, and the mixture was incubated in the dark at 25 °C for 30 min. Absorbance was measured at 517 nm, and sample blanks (extract + methanol without DPPH) were used to correct for extract background absorbance.

For the ABTS assay, the ABTS working solution was prepared by mixing 7 mM ABTS with 2.45 mM potassium persulfate and incubating for 16 h. Before use, the solution was diluted with phosphate-buffered saline (PBS, pH 7.4) to an absorbance of 0.70 ± 0.02 at 734 nm. A 100 µL aliquot of extract was added to 3.9 mL of ABTS solution and reacted for 6 min at 25 °C. Absorbance was recorded at 734 nm, and blank correction was performed using extract mixed with PBS.

Hydroxyl radical scavenging activity was measured by the Fenton reaction coupled with salicylic acid derivatization. Briefly, 0.5 mL of sample extract (appropriate concentrations) was mixed with 0.5 mL FeSO_4_ (0.9 mM) and 0.5 mL H_2_O_2_ (3 mM); then 1.0 mL salicylic acid (6 mM) was added to a final volume of 3.0 mL. The mixture was incubated at 37 °C for 30 min in the dark. Absorbance was measured at 510 nm. Sample blanks (sample and reaction mixture without H_2_O_2_) were used to correct for extract background.

Superoxide radical scavenging was determined using the PMS-NADH-NBT system. In each assay, 0.5 mL phosphate buffer (pH 7.4), 0.5 mL NBT (156 µM), 0.5 mL NADH (468 µM) and 0.4 mL sample were mixed and the reaction initiated by adding 0.1 mL PMS (60 µM). After incubation at 25 °C for 5 min, absorbance was read at 560 nm. Sample blanks (without PMS) were used to correct for sample absorbance.

### 2.6. Enzyme Inhibitory Activity Evaluations

Tyrosinase inhibitory activity was measured using L-DOPA as substrate [47]. Briefly, reactions were performed in 96-well plates in a final volume of 200 µL containing 50 µL of 50 mM phosphate buffer (pH 6.8), 25 µL of sample solution (various concentrations), 50 µL of 2.0 mM L-DOPA, and 75 µL of mushroom tyrosinase solution (final activity = 100 U/mL). The reaction mixtures were incubated at 25 °C for 10 min and absorbance was read at 475 nm. Sample blanks (sample + buffer + L-DOPA, no enzyme) were used to correct for background absorbance. Kojic acid was used as a positive control. Tyrosinase inhibition (%) was calculated relative to enzyme-only controls (no sample).

Xanthine oxidase inhibitory activity was determined by monitoring uric acid formation at 295 nm [48]. Reactions (200 µL final volume) contained 120 µL of 50 mM phosphate buffer (pH 7.5), 20 µL of sample solution, 20 µL of xanthine solution (final conc. 0.1 mM), and 40 µL of xanthine oxidase solution (final activity = 0.06 U/mL). Reactions were initiated by adding enzyme and incubated at 25 °C; absorbance at 295 nm was recorded after 5 min (or initial linear rate was used). Sample blanks (sample + buffer + xanthine, no enzyme) were used to correct for extract absorbance. Allopurinol was used as a positive control. Inhibition (%) was calculated relative to enzyme-only controls using the corresponding rate or absorbance values.

### 2.7. Essential Oil Profiling

Essential oils were dried over anhydrous Na_2_SO_4_ and diluted 1:100 (*v*/*v*) in n-hexane prior to analysis. GC-MS analysis was performed on an Agilent 7890A GC with an HP-5MS column (30 m × 0.25 mm × 0.25 μm) (Agilent Technologies, Shanghai, China). Injector temperature was set to 250 °C, injection volume 1.0 μL, and split ratio 20:1. The oven program ran from 50 °C to 250 °C at 5 °C/min; helium was used as carrier gas at 1.0 mL/min. Mass spectrometry used electron ionization (EI) at 70 eV, ion source temperature 230 °C, transfer line 280 °C, and MS scan range *m*/*z* 35–500 with a solvent delay of 3.0 min. Compounds were identified by comparison with NIST spectral library and by calculating Kovats retention indices using a C7–C30 n-alkane series. Relative abundances are reported as percent of total ion current (no internal standard was used). The Methods section has been updated with these details.

### 2.8. Data Processing and Statistical Evaluation

All experiments were conducted using three independent biological replicates, meaning that each fermentation time point (0, 2, 4, 6, 8, and 10 days) was obtained from separate, individually inoculated fermentation flasks. For each biological replicate, all chemical analyses (phenolics, flavonoids, polysaccharides, antioxidant activities, and enzyme inhibition assays) were measured in triplicate analytical determinations, and the average values were used for statistical analysis. This approach ensures biological independence while minimizing measurement variability. Statistical analyses were performed using SPSS software (version 25.0, IBM Corp., Armonk, NY, USA). Before conducting one-way ANOVA, the data were assessed for normality using the Shapiro–Wilk test and for homogeneity of variances using Levene’s test. Both assumptions were met for all datasets. Post hoc comparisons among means were carried out using Tukey’s test at a significance level of *p* < 0.05.

## 3. Results

### 3.1. Dynamics of Active Component Accumulation During SSF

**Figure 1:**
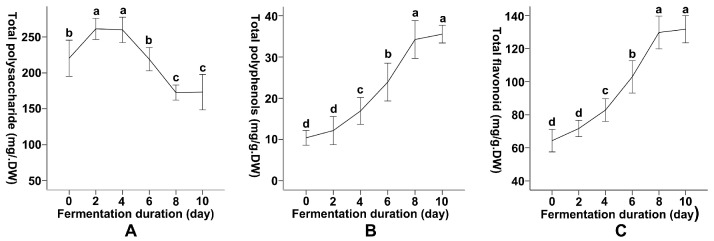
Dynamic Changes in Total Polysaccharide, Phenolic, and Flavonoid Contents of Artemisia argyi Leaves During Solid-State Fermentation by Rhizopus oryzae. Changes in (**A**) total polysaccharides, (**B**) total polyphenols, and (**C**) total flavonoids in Artemisia argyi leaves during solid-state fermentation by Rhizopus oryzae. Samples were collected at 0, 2, 4, 6, 8, and 10 days of fermentation, and the contents of the three bioactive components were quantified on a dry-weight basis. Distinct letters on the error bars in line graphs signify statistically significant differences in active component levels over varying fermentation times (*p* = 0.05).

The effects of *R*. *oryzae* SSF on the content of active compounds in *A*. *argyi* leaves were evaluated at different fermentation times (0, 2, 4, 6, 8, and 10 days) and contrasted with fresh, dried, and aged leaves (Table 1). Total polysaccharide content exhibited a dynamic response, peaking at 261.39 ± 7.09 mg/g dry weight (DW) on day 2, representing an 18.58% increase from the initial level of 220.43 ± 11.64 mg/g·DW (Figure 1A). Subsequently, a decline was observed, with values decreasing to 172.64 ± 5.13 mg/g·DW by day 8 and stabilizing at 173.26 ± 11.23 mg/g·DW by day 10 (Figure 1A). This reflected a net decrease of 21.20% in total polysaccharide content on day 8 compared to day 6. In comparison, fresh dried leaves contained 225.10 ± 10.56 mg/g·DW. However, the aged leaves exhibited the highest total polysaccharide content at 284.54 ± 12.26 mg/g·DW, significantly exceeding all fermented samples.

Table 1 exhibits a gradual increase in total polyphenol content over fermentation duration. Starting from 10.42 ± 0.85 mg/g·DW at Day 0, the content rose to 12.17 ± 1.70 mg/g·DW by Day 2 (a 16.79% increase) and continued to rise, reaching a maximum of 35.56 ± 0.98 mg/g·DW by Day 10 (Figure 1B). This represented a cumulative increase of 241.46% compared to Day 0. The highest increases occurred between Day 4 (16.93 ± 1.59 mg/g·DW, 39.11% increment) and Day 8 (34.25 ± 2.24 mg/g·DW, 43.13% increment), indicating the maximum accumulation rates during this period. The polyphenol content in fresh dried leaves (10.28 ± 0.84 mg/g·DW) was significantly lower than that in leaves fermented for 10 days and aged leaves (50.55 ± 5.44 mg/g·DW). Moreover, the total polyphenol content in aged leaves remarkably exceeded that in fermented samples.

Similarly to the total polyphenol content, the total flavonoid content also exhibited a comparable upward trend. Beginning at 64.34 ± 3.02 mg/g·DW on day 0 of fermentation, it increased to 71.69 ± 2.46 mg/g·DW by day 2 (an 11.42% increase) (Figure 1C). On day 10 of fermentation, the level reached a maximum of 131.70 ± 4.09 mg/g·DW, representing a cumulative increase of 104.67% compared to the onset value (Figure 1C). Marked increases in total flavonoid content were recorded between day 6 (102.89 ± 4.86 mg/g·DW, 24.17% increase) and day 8 (129.73 ± 4.78 mg/g·DW, 26.09% increase), indicating a sustained upward trend. The total flavonoid content in fresh dried leaves was 63.58 ± 5.17 mg/g·DW, while aged leaves exhibited the highest content of this compound at 218.56 ± 7.03 mg/g·DW, significantly higher than other leaf samples.

The variation in total polysaccharide, total polyphenol, and total flavonoid content suggests that these components typically differed significantly across various fermentation periods, particularly between days 2 and 8 of fermentation. The data also showed that although SSF with *R. oryzae* significantly improved polyphenol and flavonoid content over time, polysaccharide content reached a maximum in the early fermentation stages but subsequently declined. Together, these patterns suggest that early-stage increases in polysaccharides may result from fungal enzymatic swelling and partial solubilization of structural carbohydrates, whereas the subsequent decline is consistent with their depolymerization and utilization by *R. oryzae*. In contrast, the gradual accumulation of phenolics and flavonoids likely reflects the progressive release of bound phenolic conjugates and the activation of phenylpropanoid-related metabolic pathways during fermentation.

### 3.2. Temporal Evolution of Antioxidant Capacity During SSF

**Figure 2:**
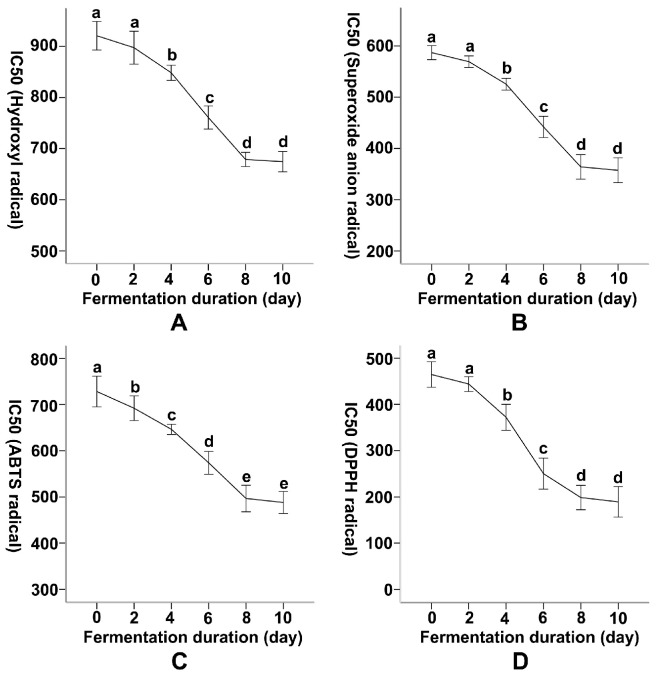
Dynamic Changes in the Antioxidant Capacities of Artemisia argyi Leaves During Solid-State Fermentation by Rhizopus oryzae. Changes in IC50 values of antioxidant activities of Artemisia argyi leaf extracts during solid-state fermentation by Rhizopus oryzae. (**A**) Hydroxyl radical scavenging activity, (**B**) superoxide anion radical scavenging activity, (**C**) ABTS radical cation scavenging activity, and (**D**) DPPH radical scavenging activity. IC50 values were measured at different fermentation durations (0, 4, 6, 8, and 10 days), with lower IC50 values indicating stronger antioxidant activity. Distinct letters on the error bars in line graphs signify statistically significant differences in antioxidant capacities over varying fermentation times (*p* = 0.05).

The antioxidant capacity of *A. argyi* leaves was evaluated by determining the IC50 values for scavenging reactive oxygen species (ROS), including hydroxyl radicals, superoxide anion radicals, ABTS radicals, and DPPH radicals. Monitoring was conducted during the duration of SSF of *A. argyi* leaves by *R. oryzae* (0, 2, 4, 6, 8, and 10 days) and compared with fresh dried and aged leaves (Table 2). The IC50 value of *A. argyi* leaves for hydroxyl radicals at Day 0 was 920.80 ± 14.00 μg/mL. The IC50 values for superoxide anion radicals, ABTS, and DPPH radicals were 586.94 ± 6.13 μg/mL, 728.19 ± 16.00 μg/mL, and 464.60 ± 13.65 μg/mL, respectively. As fermentation time increased, the IC50 values of fermented *A. argyi* gradually decreased (Figure 2). The IC50 against hydroxyl radicals dropped to 674.53 ± 9.54 μg/mL on day 10 (a 26.75% reduction compared to day 0). The most significant IC50 decrease occurred between Day 6 (760.85 ± 10.44 μg/mL) and Day 8 (678.82 ± 6.43 μg/mL) (Figure 2A). The IC50 for superoxide anion radicals decreased to 357.46 ± 12.08 μg/mL on day 10 (a 39.09% reduction), with the most pronounced decrease occurring between days 6 and 8, from 442.11 ± 9.59 μg/mL to 364.08 ± 11.32 μg/mL (Figure 2B).

Like for the scavenging of hydroxyl radicals and superoxide anion radicals, the IC50 value for ABTS radicals in fermented *A. argyi* leaves also reached its lowest point on day 10. This value (487.91 ± 11.89 μg/mL) represented a 33.01% reduction compared to the prefermentation level (728.19 ± 16.00 μg/mL) (Figure 2C). The most pronounced decrease occurred between day 6 (573.69 ± 11.64) and day 8 (496.54 ± 14.22 μg/mL, −11.23%). The IC50 against DPPH radicals showed the most notable improvement during fermentation. It decreased from 464.60 ± 13.65 μg/mL on day 0 to 189.26 ± 15.87 μg/mL on day 10 (a 59.26% reduction) (Figure 2D). The most rapid decrease occurred from day 4 (372.01 ± 12.65 μg/mL) to day 6 (250.22 ± 16.33 μg/mL, −20.65%) (Figure 2D). The IC50 values of fresh dried leaves for various reactive oxygen species did not differ significantly from those of fermented *A. argyi* leaves on day 0. However, the IC50 values for both fresh dried leaves and fermented leaves were significantly higher than those of aged *A. argyi* leaves, suggesting that aged *A. argyi* leaves exhibit notably superior performance compared to the other two.

These observations demonstrate that SSF with *R. oryzae* progressively strengthened the antioxidant capacity of *A. argyi* leaves. Optimal ROS scavenging effects were observed between days 4 and 8, indicating the most rapid improvement during this interval; however, the absolute lowest IC50 values were achieved on day 10. Although fermentation enhanced the leaves’ antioxidant effects, aged leaves still exhibited superior antioxidant activity, possibly due to biochemical transformations occurring over an extended period.

Across fermentation, the IC50 values of the antioxidant assays showed a 0.27–0.59-fold decreases, broadly paralleling the 1.05–2.41-fold increases observed in total phenolics and flavonoids. These proportional changes are consistent with a phenolic-driven enhancement of antioxidant activity.

### 3.3. Progressive Enhancement of Enzyme Inhibitory Activities During SSF

**Figure 3:**
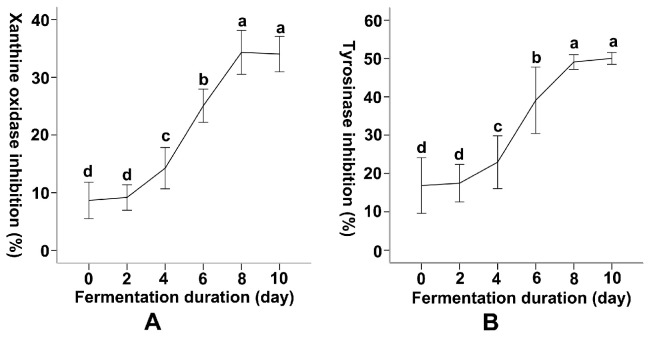
Dynamic Changes in the Enzyme Inhibitory Activity of Artemisia argyi Leaves During Solid-State Fermentation by Rhizopus oryzae. Changes in xanthine oxidase inhibition and tyrosinase inhibition percentages in Artemisia argyi leaves during solid-state fermentation by Rhizopus oryzae. (**A**) Xanthine oxidase inhibition percentages, (**B**) tyrosinase inhibition percentages. Inhibition percentages were measured at different fermentation durations (0, 4, 6, 8, and 10 days). Distinct letters on the error bars in line graphs signify statistically significant differences in enzyme inhibitory over varying fermentation times (*p* = 0.05).

Xanthine oxidase and tyrosinase are key enzymes closely associated with gout and skin pigmentation, respectively. The inhibition rates of these two enzymes by fermented *A*. *argyi* leaf extract were assessed across different SSF durations (0, 2, 4, 6, 8, and 10 days) of *R*. *oryzae* and were evaluated against fresh dried and aged leaves (Table 3). The initial inhibition rate of xanthine oxidase at an *A*. *argyi* leaf concentration of 100 μg/mL was minimal at Day 0 (8.69 ± 1.38%), slightly increasing to 9.17 ± 1.04% at Day 2 (a 5.52% increase). A significant increase was observed starting from day 4 (14.25 ± 1.77%, a 55.40% increase compared to day 2), reaching a maximum of 34.33 ± 1.90% on day 8, followed by a slight decrease to 34.04 ± 1.43% on day 10 (−0.84%) (Figure 3A). The inhibition rate of fresh dried leaves was 8.74 ± 1.93%, while aged leaves exhibited the highest inhibitory activity at 43.97 ± 2.51%, significantly exceeding that of the 10-day fermented sample.

Fermented *A. argyi* leaves exhibited a tyrosinase inhibition trend similar to that of xanthine oxidase. The inhibition rate started at 16.84 ± 3.53% on Day 0, gradually increasing to 17.46 ± 2.38% by Day 2 (a 3.68% increase) (Figure 3B). The most pronounced increase in tyrosinase inhibition occurred between day 4 and day 6, corresponding to the largest percentage increment (70.40%) shown in Table 3, whereas the changes between other consecutive intervals were comparatively smaller (Figure 3B). Maximum inhibition was reached on day 10 (50.02 ± 0.76%). Fresh dried leaves show an inhibition rate of 16.43 ± 1.67%, while aged leaves reached a peak of 55.99 ± 1.90%, significantly exceeding both fresh dried leaves and samples fermented for 10 days.

These findings suggest that SSF by *R. oryzae* enhances the inhibitory potential of *A. argyi* leaves against xanthine oxidase and tyrosinase. The inhibitory activity against both enzymes increased most rapidly during SSF between days 6 and 8, while xanthine oxidase inhibition showed a slight decline from day 8 to day 10. Aged leaves still exhibited superior inhibitory efficacy; this may be attributed to prolonged processes.

It should be noted that enzyme inhibition was evaluated only at a single testing dose (100 μg/mL), which restricts the quantitative comparison of inhibitory strength among treatments. Future determination of IC50 values or dose–response curves for tyrosinase and xanthine oxidase would allow for a more precise assessment of the contributions of fermentation-induced phenolic enrichment to enzyme inhibition.

### 3.4. Fungal-Induced Modulation of Volatile Compounds in A. argyi Essential Oils

Gas chromatography-mass spectrometry (GC-MS) analysis revealed distinct patterns in the volatile constituents of *A*. *argyi* leaves across treatments, as detailed in Table 4. Oxygenated monoterpenes (OMTs) were the predominant constituents, accounting for 45.55%, 38.85%, and 50.56% of the fermented, control, and aged samples, respectively. Olefins and their derivatives (ODs) represented the second largest group, comprising 9.58% in fermented, 11.77% in control, and 7.26% in aged leaves. Aromatic compounds (ACs), monoterpenes (MTs), sesquiterpenes (STs), and oxygenated sesquiterpenes (OSTs) contributed lesser proportions, ranging from 6.93 to 7.94%, 6.69–7.40%, 3.66–5.69%, and 0.76–1.32%, respectively.

R. oryzae-mediated SSF led to a reconfiguration of the volatile spectrum, elevating OMT levels while diminishing OD, ST, and AC fractions compared to control leaves. This pattern was also partially mirrored in aged samples. Essential oil yield increased to 7.40 ± 0.99 mg/g·DW in fermented leaves from 4.70 ± 0.71 mg/g·DW in controls, though remaining below the 12.50 ± 0.77 mg/g·DW in aged leaves. Compared to the accumulation observed during the aging process, the increase in OMT under SSF conditions suggests that *R. oryzae* biotransformation selectively promotes the retention of specific volatiles.

Notable component-specific shifts included eucalyptol (OMT), which rose to 22.27 ± 1.55% in fermented leaves compared to the control group (17.18 ± 1.46%) and reached 24.37 ± 1.93% in aged samples. Borneol and DL-menthol also showed elevated relative abundances in both processed groups, with values of 1.10 ± 0.23% and 6.27 ± 0.10% in fermented leaves, respectively. Conversely, α-phellandrene (MT) declined from 3.18 ± 0.08% in controls to 2.29 ± 0.21% in fermented and 0.89 ± 0.21% in aged leaves. Compounds such as isoanethole (AC) and cis-anethol (AC) were either newly detected or absent in the fermented samples, reflecting treatment-specific metabolic changes. Identifications were confirmed using Kovats retention indices and molecular weights, with GC capturing approximately 75% of the total composition due to its selective detection of volatile compounds.

Alterations in essential oil components during SSF suggest that *R*. *oryzae* fermentation is consistent with targeted enrichment of specific volatiles. This may enhance the functional properties of *A. argyi* beyond mere yield increases.

It should be noted that the essential oil profiles were analyzed only on three selected samples: the fresh-dried control, the 10-day fermented sample, and the three-year aged leaves. Thus, the temporal dynamics of volatile changes throughout the entire SSF process remain unknown. Although the 10-day fermented sample provides a representative endpoint of fungal biotransformation, the absence of intermediate time point analyses limits our understanding of how key volatile constituents evolve during fermentation. Because only endpoint and control samples were evaluated, the study cannot determine whether key volatiles accumulate gradually, appear abruptly at later stages, or fluctuate during SSF. Future work incorporating multiple intermediate sampling points would allow for a more complete characterization of SSF-induced modifications in the volatile metabolome of *A. argyi*.

## 4. Discussion

This investigation demonstrates that SSF using *R. oryzae* significantly enhanced the content of bioactive compounds and in vitro biological activity in *A. argyi* leaves. Although this study did not directly compare SSF with LSF on *A. argyi*, the observed enhancements in bioactive compounds and functional activities are consistent with literature reports that highlight the advantages of SSF in herbal processing [21,49]. Therefore, SSF may represent a potentially promising alternative to conventional LSF approaches, pending further direct comparative studies. Unlike LSF, which relies on high moisture content, SSF operates under low moisture conditions that more closely mimic natural microbial environments [17,20,50]. These fermentation conditions allow for higher concentrations of microbial metabolites within the substrate and simplify operational requirements [17,51,52]. Our findings indicate a noteworthy rise in total polyphenols (increasing from 10.42 ± 0.85 mg/g·DW to 35.56 ± 0.98 mg/g·DW over 10 days) and flavonoids (rising from 64.34 ± 3.02 mg/g·DW to 131.70 ± 4.09 mg/g·DW), accompanied by enhanced antioxidant capacity (e.g., DPPH IC50 decreased from 464.60 ± 13.65 μg/mL to 189.26 ± 15.87 μg/mL) and enhanced enzyme inhibitory activity (inhibition rate of xanthine oxidase by fermented *A. argyi* leaves at 100 μg/mL reached 34.04 ± 1.43%). In LSF, dilution of microbial metabolites may limit the accumulation of target compounds undergoing conversion [53,54,55]. Unlike LSF, the solid substrate in SSF allows fungal mycelium to penetrate plant tissues and cells, enabling more efficient enzymatic hydrolysis while avoiding shear stress, which is believed to degrade sensitive compounds in agitated liquid systems [20,22,56]. Because no direct LSF comparison was performed, our inference relies on typical values reported in the literature, which suggest that the improvements observed under SSF may reflect relatively efficient substrate conversion. However, this remains a comparative indication rather than experimental evidence.

A key advantage of SSF over LSF lies in its superior extraction and concentration of bioactive compounds. The increase in phenolic content is likely attributable to hydrolytic enzymes such as β-glucosidase. Although this explanation is inferential, previous studies have shown that *R. oryzae* ATCC 34612 produces β-glucosidase and other cell-wall-degrading enzymes, which is consistent with the phenolic enhancement observed here [37,57,58]. These enzymes efficiently cleave glycosidic bonds and release bound polyphenolic compounds from the plant cell matrix [58,59]. This enzymatic profile is particularly suited for solid substrates such as herbal leaves and rhizomes, where microbial penetration into the matrix enables targeted biotransformation. In contrast, LSF typically yields lower outputs due to enzyme and substrate dilution in aqueous media, necessitating energy-intensive concentration steps like additional centrifugation or evaporation [51,60,61]. Our findings clearly demonstrate progressively enhanced antioxidant activity during SSF of *A. argyi*. While literature often reports lower or less sustained improvements during LSF of other plant materials [51,62], these comparisons derive from external studies. Because no LSF treatment was included here, our data do not establish SSF superiority for *A. argyi*; rather, they indicate that SSF was effective under the specific conditions tested. Furthermore, the time-course dynamics in our study indicate that most bioactive compounds peak between days 6 and 8, highlighting SSF’s efficiency in short term processes. This avoids the extended incubation times typically required in LSF to compensate for diffusion limitations [51,62,63].

The early peak in polysaccharide content observed on day 2 followed by a pronounced decline toward day 8 can be biologically explained by the metabolic dynamics of *R. oryzae*. During the initial stages of SSF, the fungus secretes hydrolytic enzymes that rapidly break down cell-wall polysaccharides into smaller, water-soluble fractions, leading to a transient increase in extractable polysaccharides [63]. As fermentation progresses, these newly liberated soluble sugars become readily accessible carbon sources that fuel mycelial proliferation and energy metabolism. Consequently, *R. oryzae* is likely to consume a portion of these soluble polysaccharides during its exponential growth phase (typically between days 2 and 6), resulting in the gradual decline observed by day 8. This pattern is consistent with reports that filamentous fungi preferentially utilize easily metabolizable sugars after initial hydrolysis, leading to a biphasic profile of polysaccharide liberation followed by fungal assimilation [64,65,66]. Additionally, the decline may also reflect the shift from primary metabolism to secondary metabolite production, during which fungal carbon demand increases, and formerly accumulated carbohydrates are redirected into metabolic pathways supporting enzyme synthesis and sporulation.

SSF offers distinct advantages by creating a microenvironment that enhances antioxidant activity while preventing the loss of such activity often observed in LSF. In this study, the radical scavenging capacity of fermented *A. argyi* leaves was markedly enhanced, as evidenced by changes in the IC50 values for fermented *A. argyi* against various oxygen radicals. For instance, the IC50 values decreased for hydroxyl radicals (from 920.80 ± 14.00 μg/mL to 674.53 ± 9.54 μg/mL), superoxide anion radicals (from 586.94 ± 6.13 μg/mL to 357.46 ± 12.08 μg/mL), and ABTS radicals (from 728.19 ± 16.00 μg/mL to 487.91 ± 11.89 μg/mL). The enhanced antioxidant activity may stem from the synergistic release of flavonoids and phenolic compounds, which act as effective electron donors in free radical quenching assays [67,68,69]. This advantage stems from SSF minimizing volatile losses and promoting heightened metabolite-enzyme interactions without dilution effects, thus raising the inhibition threshold in submerged cultures. This finding is supported by prior research. The research indicated that the IC50 values of SSF substrates are significantly lower than those of LSF substrates. Our findings are consistent with this, as the gradual decrease in IC50 during fermentation correlates with polyphenol accumulation, whereas this trend is less pronounced in LSF [70,71]. Due to the limitations of our antioxidant activity experiments, this indicates that the potential of fermented *A. argyi* leaves as antioxidant supplements needs to be confirmed in vivo.

SSF effectively enhances enzyme inhibitory activity, offering advantages for potential therapeutic applications that are difficult to achieve with LSF. The enhanced tyrosinase inhibition rate (up to 50.02 ± 0.76% at 100 μg/mL) and xanthine oxidase inhibition rate (34.04 ± 1.43%) in fermented *A. argyi* leaves make solid-state fermented leaves potential candidates against cosmetic/dermatological and hyperuricemia-related applications, respectively. In LSF, highly mobile liquids typically dilute inhibitory enzymes, reducing their efficacy, whereas SSF environments promote aggregation and concentration of inhibitory enzymes, leading to elevated inhibition rates in comparative studies [72,73,74]. This trend is also reflected in our findings, where inhibition peaked between days 6 and 8.

In the present study, only three points, the fresh dried leaves, aged leaves, and the 10-day SSF samples, were subjected to GC-MS analysis. The SSF-treated leaves showed higher relative levels of borneol and eucalyptol compared with the fresh dried samples, and these compounds have been previously associated with anti-inflammatory and antibacterial potential in the literature [75,76,77]. While these compositional changes may be favorable for the functional quality of *A. argyi*, the current data do not allow us to determine how these volatiles evolved throughout the fermentation process. Furthermore, because no LSF treatment was included, we cannot conclude whether such enrichment patterns are unique to SSF. The findings therefore indicate only that SSF, under the conditions tested, preserved or enhanced selected volatiles relative to non-fermented controls, warranting further investigation of their biological and processing implications.

From a sustainability perspective, SSF offers significant advantages over LSF, aligning with eco-friendly bioprocessing objectives. Due to the natural barrier provided by solid substrates, SSF requires lower water consumption (typically < 1:2 substrate to liquid ratio, compared to 1:10–20 for LSF) and reduced risk of bacterial contamination, making it a stable, low cost, and sustainable option for herbal fermentation [51,62,78]. Previous studies have reported that SSF generally requires lower moisture levels and may consume 30–50% less energy than LSF, largely due to reduced aeration, agitation, and heating demands [51,79,80]. These sustainability benefits, however, are literature-based expectations and were not assessed in the present study. Our results demonstrate only that SSF effectively enhanced the bioactive components of *A. argyi* within the tested timeframe. While such improvements are consistent with the notion that SSF can achieve desirable biochemical transformations with potentially lower resource inputs, direct evaluation of energy use or carbon footprint would be necessary to validate these advantages in our system. Economically, SSF using herbal substrates like *A. argyi* presents an ideal opportunity for industrial herbal fermentation production. By simplifying equipment and reducing energy consumption, it holds promise for significantly lowering costs. In contrast, the high operational expenses associated with medium preparation and sterilization in LSF limit its practicality for small-scale or rural cooperative settings.

Compared with the multi-year aging process, which resulted in the highest levels of polyphenols, flavonoids, and antioxidant activity, SSF achieved substantial, though still comparatively lower, enhancements within just a few days, indicating that SSF can partially mimic the biochemical trajectory of aging but does not reach the same magnitude of transformation. The divergence between SSF-derived and aged-leaf profiles suggests that different biochemical pathways, microbial enzymatic hydrolysis versus slow oxidative/structural transformations, drive the observed changes. Therefore, SSF should be viewed as a complementary short-term processing strategy that produces a distinct biochemical profile rather than a direct substitute for multi-year aging.

Several limitations of this study should be acknowledged. First, no LSF experiments on *A. argyi* were performed, and all comparisons with LSF are therefore derived solely from published literature rather than from direct experimental evidence. Second, although increases in phenolics, flavonoids, polysaccharides, and bioactivities were observed, the underlying mechanisms were not investigated; no enzyme activity assays (e.g., β-glucosidase, cellulase, or other hydrolases) were performed to confirm the biochemical pathways responsible for these changes. Third, the study utilized a single fungal strain (*R. oryzae* ATCC 34612) and a single batch of *A. argyi* leaves, which may limit the generalizability of the findings across cultivars, seasons, or microbial populations. Finally, all functional evaluations, including antioxidant assays and enzyme inhibition, were conducted exclusively in vitro, and thus the biological relevance and efficacy of the fermented extracts remain to be validated in cellular or in vivo systems.

## 5. Conclusions

SSF of *A. argyi* leaves by *R*. *oryzae* significantly enhanced their phytochemical content and biological activity within a short fermentation period. The synergistic increase in phenolic and flavonoid compounds was accompanied by decreased IC50 values for active oxidants and elevated inhibition rates for xanthine oxidase and tyrosinase. These enhancements are consistent with fungal enzyme-mediated hydrolysis contributing to the release of bound phenolics, although this remains speculative because no enzyme assays or bound/free phenolic analyses were performed. Volatile compound analysis further confirmed the selective enrichment of oxygenated monoterpenes. These findings demonstrate that SSF increases the quantity of bioactive compounds and modulates their composition. Compared to the several years of aging time required for aged *A. argyi* leaves, SSF leaves achieved substantial biochemical enhancement within 10 days, though still lower than that of 3-year aged leaves. Thus, SSF should be interpreted as an efficient short-term processing method that yields partially improved phytochemical profiles, rather than a replacement for multi-year aging. Furthermore, SSF offers significant sustainability benefits over LSF, including reduced water and energy consumption, decreased wastewater generation, and lower carbon emissions. *R. oryzae*-mediated SSF has potential as a green, low-cost, and scalable bioprocess for enhancing the pharmacological qualities and nutritional potential of herbal resources. Future work involving mechanistic enzyme analyses, comparison with liquid-state fermentation, and multi-batch validation will be needed to determine the specific contexts in which SSF can complement or enhance traditional processing approaches.

## Figures and Tables

**Table 1:** Alterations in the content of active components in various solid-state fermentation durations of *Artemisia argyi* leaves.

Leaf Fermentation Duration (Day)	Total Polysaccharide (mg/g·DW)	Total Polyphenols (mg/g·DW)	Total Flavonoid (mg/g·DW)
0	220.43 ± 11.64 ^b^	10.42 ± 0.85 ^d^	64.34 ± 3.02 ^d^
2	261.39 ± 7.09 ^a^ (18.58)	12.17 ± 1.70 ^d^ (16.79)	71.69 ± 2.46 ^d^ (11.42)
4	259.87 ± 8.71 ^a^ (−0.58)	16.93 ± 1.59 ^c^ (39.11)	82.86 ± 3.42 ^c^ (15.58)
6	219.10 ± 7.65 ^b^ (−15.69)	23.93 ± 2.16 ^b^ (41.35)	102.89 ± 4.86 ^b^ (24.17)
8	172.64 ± 5.13 ^c^ (−21.20)	34.25 ± 2.24 ^a^ (43.13)	129.73 ± 4.78 ^a^ (26.09)
10	173.26 ± 11.23 ^c,C^ (0.36)	35.56 ± 0.98 ^a,B^ (3.82)	131.70 ± 4.09 ^a,B^ (1.52)
Fresh dried leaves	225.10 ± 10.56 ^B^	10.28 ± 0.84 ^C^	63.58 ± 5.17 ^C^
Aged leaves	284.54 ± 12.26 ^A^	50.55 ± 5.44 ^A^	218.56 ± 7.03 ^A^

Mean ± SD from three independent biological replicates. Values in parentheses indicate the percentage increase relative to the preceding fermentation time (negative values indicate decreases). Values followed by different lowercase letters within a column differ significantly among fermentation times, while different uppercase letters indicate significant differences among the 10 days fermentation, fresh dried leaves, and aged leaves (*p* < 0.05).

**Table 2:** IC50 of *Artemisia. argyi* leaves in reactive oxygen species scavenging under various solid-state fermentation durations.

Leaf Fermentation Duration (Day)	IC50 (Hydroxyl Radical)	IC50 (Superoxide Anion Radical)	IC50 (ABTS Radical)	IC50 (DPPH Radical)
0	920.80 ± 14.00 ^a^	586.94 ± 6.13 ^a^	728.19 ± 16.00 ^a^	464.60 ± 13.65 ^a^
2	897.42 ± 16.04 ^a^ (−2.54)	569.26 ± 5.59 ^a^ (−3.01)	692.01 ± 12.53 ^b^ (−4.97)	443.99 ± 7.95 ^a^ (−4.44)
4	848.34 ± 7.14 ^b^ (−5.47)	525.33 ± 5.26 ^b^ (−7.72)	646.24 ± 5.29 ^c^ (−6.61)	372.01 ± 12.65 ^b^ (−16.21)
6	760.85 ± 10.44 ^c^ (−10.31)	442.11 ± 9.59 ^c^ (−15.84)	573.69 ± 11.64 ^d^ (−11.23)	250.22 ± 16.33 ^c^ (−32.74)
8	678.82 ± 6.43 ^d^ (−10.78)	364.08 ± 11.32 ^d^ (−17.65)	496.54 ± 14.22 ^e^ (−13.45)	198.54 ± 13.05 ^d^ (−20.65)
10	674.53 ± 9.54 ^d,B^ (−0.63)	357.46 ± 12.08 ^d,B^ (−1.82)	487.91 ± 11.89 ^e,B^ (−1.74)	189.26 ± 15.87 ^d,B^ (−4.67)
Fresh dried leaves	920.75 ± 9.52 ^A^	577.62 ± 7.54 ^A^	727.76 ± 6.86 ^A^	472.21 ± 13.33 ^A^
Aged leaves	380.16 ± 8.59 ^C^	303.28 ± 21.50 ^C^	319.98 ± 6.86 ^C^	63.28 ± 4.75 ^C^

Each value represents the mean ± SD of triplicate independent biological assays. Values in parentheses indicate the percentage change relative to the preceding fermentation time (negative values indicate decreases in IC50). Values followed by different lowercase letters within a column differ significantly among fermentation times, while different uppercase letters indicate significant differences among the 10-day fermentation leaves, fresh dried leaves, and aged leaves (*p* < 0.05).

**Table 3:** Inhibition (%) of Xanthine Oxidase and Tyrosinase by *Artemisia argyi* leaves at different solid-state fermentation times.

Leaf Fermentation Duration (Day)	Xanthine Oxidase Inhibition (%) at 100 μg/mL	Tyrosinase Inhibition (%) at 100 μg/mL
0	8.69 ± 1.38 ^d^	16.84 ± 3.53 ^d^
2	9.17 ± 1.04 ^d^ (5.52)	17.46 ± 2.38 ^d^ (3.68)
4	14.25 ± 1.77 ^c^ (55.40)	22.94 ± 3.19 ^c^ (31.39)
6	25.07± 1.44 ^b^ (75.93)	39.09 ± 4.05 ^b^ (70.40)
8	34.33 ± 1.90 ^a^ (36.94)	49.07 ± 0.95 ^a^ (25.53)
10	34.04 ± 1.43 ^a,B^ (−0.84)	50.02 ± 0.76 ^a,B^ (1.94)
Fresh dried leaves	8.74 ± 1.93 ^C^	16.43 ± 1.67 ^C^
Aged leaves	43.97 ± 2.51 ^A^	55.99 ± 1.90 ^A^

Data represent the average ± standard deviation of three separate biological experiments. Values in parentheses indicate the percentage increase relative to the preceding fermentation time. Values followed by different lowercase letters within a column differ significantly among fermentation times, while different uppercase letters indicate significant differences among the 10-day fermentation leaves, fresh dried leaves, and aged leaves (*p* < 0.05).

**Table 4:** Composition and relative contents of essential oil in *A. argyi* leaves under various treatments.

	Common Name	Systematic Name	Chemical Formula	Retention Index	Molecular Weight	Fermented Leaves (%)	Control Leaves (%)	Aged Leaves (%)
MT	α-pinene	2,6,6-trimethyl-bicyclo[3.1.1-2]heptene	C_10_H_16_	942	136.23	0.36 ± 0.03 ^c^	0.46 ± 0.02 ^b^	0.65 ± 0.01 ^a^
	camphene	2,2-Dimethyl-3-methylene-bicyclo[2.2.1]heptane	C_10_H_16_	951	136.23	0.78 ± 0.04 ^b^	0.74 ± 0.04 ^b^	1.20± 0.06 ^a^
	trans-sabinene	(1R)-4-methylene-1-(1-methylethyl)-bicyclo[3.1.0]hexane	C_10_H_16_	963	136.23	1.29 ± 0.32 ^a^	1.58 ± 0.23 ^a^	1.23 ± 0.13 ^a^
	(+)-2-carene	(1S,6R)-3,7,7-Trimethylbicyclo[4.1.0]hept-2-ene	C_10_H_16_	990	136.23	0.68 ± 0.17 ^b^	0.77 ± 0.10 ^b^	1.16 ± 0.29 ^a^
	α-phellandrene	2-methyl-5-(1-methylethyl)-1,3-Cyclohexadiene	C_10_H_16_	1008	136.23	2.29 ± 0.21 ^b^	3.18 ± 0.08 ^a^	0.89 ± 0.21 ^c^
	γ-terpinene	1-methyl-4-isopropyl-1,4-cyclohexadiene	C_10_H_16_	1058	136.23	1.34 ± 0.09 ^a^	0.67 ± 0.25 ^b^	1.56 ± 0.06 ^a^
Total MT						6.74	7.40	6.69
OMT	eucalyptol	1,3,3-Trimethyl-2-oxabicyclo[2.2.2]octane	C_10_H_18_O	1033	154.24	22.27 ± 1.55 ^a^	17.18 ± 1.46 ^b^	24.37 ± 1.93 ^a^
	D-camphor	7,7-trimethyl-(1theta)-bicyclo[2.2.1]heptan-2-on	C_10_H_16_O	1133	152.23	1.67 ± 0.25 ^a^	1.63 ± 0.30 ^a^	0.37 ± 0.28 ^b^
	(S)-cis-verbenol	1,2,4-trimethyl-5-[2-(2,4,5-trimethylphenyl)ethyl]benzene	C_10_H_16_O	1141	152.23	0.34 ± 0.12 ^b^	0.33 ± 0.08 ^b^	0.93 ± 0.35 ^a^
	DL-2-bornanol	(1S,4R,6R)-1,7,7-Trimethylbicyclo[2.2.1]heptan-6-ol	C_10_H_18_O	1152	154.25	4.29 ± 0.35 ^a^	3.64 ± 0.37 ^b^	4.26 ± 0.37 ^a^
	borneol	(1R,2S,4R)-rel-1,7,7-TriMethylbicyclo[2.2.1]heptan-2-ol	C_10_H_18_O	1164	154.25	1.10 ± 0.23 ^b^	0.48 ± 0.15 ^c^	3.51 ± 0.29 ^a^
	DL-menthol	(1R,2R,5S)-5-methyl-2-propan-2-yl-1-cyclohexanol	C_10_H_20_O	1169	156.27	6.27 ± 0.10 ^a^	5.36 ± 0.09 ^b^	6.74 ± 0.41 ^a^
	terpinen-4-ol	1-isopropyl-4-methyl-cyclohex-3-enol	C_10_H_18_O	1174	154.25	0.65 ± 0.10 ^ab^	0.56 ± 0.28 ^b^	0.94 ± 0.11 ^a^
	α-terpineol	1-Methyl-4-(1-hydroxy-1-methylethyl)-1-cyclohexene	C_10_H_18_O	1190	154.25	0.86 ± 0.09 ^b^	0.53 ± 0.08 ^c^	2.06 ± 0.18 ^a^
	α-terpineol	(s)-(-)-p-menth-1-en-8-o	C_10_H_18_O	1196	154.25	3.58 ± 0.26 ^b^	4.53 ± 0.32 ^a^	3.21 ± 0.32 ^b^
	trans-p-menth-1-en-3-ol	trans-3-methyl-6-(1-methylethyl)-2-cyclohexen-1-ol	C_10_H_18_O	1213	154.25	1.18 ± 0.13 ^a^	1.33 ± 0.12 ^a^	0.82 ± 0.07 ^b^
	(-)L-α-terpineol	.alpha.,.alpha.-4-trimethyl-,(S)-3-Cyclohexene-1-methanol	C_10_H_18_O	1223	154.25	0.23 ± 0.03 ^b^	0.26 ± 0.06 ^b^	0.62 ± 0.11 ^a^
	cis-carveol	(1R,5R)-2-Methyl-5-isopropenyl-2-cyclohexene-1-ol	C_10_H_16_O	1227	152.23	0.36 ± 0.03 ^a^	0.36 ± 0.05 ^a^	0.00
	carveol	2-methyl-5-(1-methylethenyl)-2-cyclohexenol	C_10_H_16_O	1235	152.23	1.58 ± 0.05 ^b^	1.42 ± 0.04 ^b^	2.15 ± 0.17 ^a^
	L(-)-perillaldehyde	(S)-4-(Prop-1-en-2-yl)-cyclohex-1-enecarbaldehyde	C_10_H_14_O	1272	150.22	1.17 ± 0.16 ^a^	1.24 ± 0.19 ^a^	0.58 ± 0.24 ^b^
Total OMT						45.55	38.85	50.56
ST	α-copaene	Tricyclo[4.4.0.02,7]dec-3-ene, 1,3-dimethyl-8-(1-methylethyl)-, stereoisomer	C_15_H_24_	1378	204.35	0.67 ± 0.11 ^a^	0.45 ± 0.06 ^b^	0.12 ± 0.04 ^c^
	β-caryophyllene	2-Methylene-6,10,10-trimethyl bicyclo[7.2.0]undec-5-ene	C_15_H_24_	1420	204.35	1.15 ± 0.19 ^a^	1.19 ± 0.24 ^a^	1.02 ± 0.08 ^a^
	trans-caryophyllene	2-Methylene-6,10,10-trimethyl bicyclo[7.2.0]undec-5-ene	C_15_H_24_	1432	204.35	2.16 ± 0.22 ^b^	3.24 ± 0.36 ^a^	1.67 ± 0.05 ^c^
	α-caryophyllene	(E,E,E)-2,6,6,9-Tetramethyl-1,4,8-cycloundecatriene	C_15_H_24_	1459	204.35	0.38 ± 0.04 ^a^	0.35 ± 0.09 ^a^	0.00
	cadina-3,9-diene	1-Isopropyl-4,7-dimethyl-1,2,4a,5,8,8a-hexahydronaphthalene-, [1S-(1α,4aβ,8aα)]-	C_15_H_24_	1519	204.35	0.50 ± 0.08 ^b^	0.46 ± 0.04 ^b^	0.85 ± 0.07 ^a^
Total ST						4.86	5.69	3.66
OST	spathulenol	(1aR,4aβ,7aα,7bβ)-Decahydro-1,1,7-trimethyl-4-methylene-1H-cycloprop[e]azulen-7α-ol	C_15_H_24_O	1577	220.35	0.18 ± 0.05 ^a^	0.16 ± 0.03 ^ab^	0.10 ± 0.03 ^b^
	caryophyllene oxide	(1r,4r,6r,10s)-9-methylene-4,12,12-trimethyl-5-oxatricyclo[8.2.0.0(4,6)]dodecane	C_15_H_24_O	1590	220.35	0.28 ± 0.05 ^a^	0.35 ± 0.06 ^a^	0.29 ± 0.12 ^a^
	eudesm-7(11)-en-4-ol	(1S,4aS,8aS)-Decahydro-1,4a-dimethyl-7-(1-methylethylidene)-1-naphthalenol	C_15_H_26_O	1683	222.36	0.68 ± 0.10 ^a^	0.81 ± 0.07 ^a^	0.37 ± 0.05 ^b^
Total OST						1.14	1.32	0.76
AC	o-cymene	1-methyl-2-(1-methylethyl)benzene	C_10_H_14_	1020	134.22	1.89 ± 0.17 ^a^	1.88 ± 0.22 ^a^	0.90 ± 0.19 ^b^
	p-Cymen-8-ol	1-Methyl-4-(1-hydroxy-1-methylethyl)benzene	C_10_H_14_O	1187	150.22	2.13 ± 0.33 ^b^	3.21 ± 0.14 ^a^	1.64 ± 0.20 ^c^
	isoanethole	1-methoxy-4-prop-2-enylbenzene	C_10_H_12_O	1201	148.20	0.00	0.50 ± 0.10 ^b^	0.80 ± 0.13 ^a^
	cis-anethol	trans-1-methoxy-4-(1-propenyl)benzene	C_10_H_12_O	1257	148.20	0.12 ± 0.05 ^b^	0.21 ± 0.03 ^a^	0.00
	1-methylnaphthalene	Methyl-1-naphthalene	C_11_H_10_	1311	142.20	0.11 ± 0.04 ^a^	0.09 ± 0.03 ^a^	0.00
	eugenol	4-allyl-2-methoxy-pheno	C_10_H_12_O_2_	1384	164.20	1.84 ± 0.32 ^ab^	2.10 ± 0.10 ^a^	1.62 ± 0.25 ^b^
	butylated hydroxytoluene	(Z)-retro-αretro-Methyl-αButylated hydroxytoluene Manufacturer	C_15_H_24_O	1508	220.35	1.45 ± 0.07 ^a^	1.36 ± 0.08 ^a^	1.52± 0.10 ^a^
	Thujopsenal	cyclopropa[d]naphthalene-2-carboxaldehyde, 1,1a,4,4a,5,6,7,8-octahydro-4a,8,8-trimethyl-	C_16_H_24_O_2_	1717	248.36	0.40 ± 0.04 ^a^	0.39 ± 0.02 ^a^	0.45 ± 0.05 ^a^
Total AC						7.94	9.74	6.93
OD	artemisia ketone	3,3,6-trimethylhepta-1,5-dien-4-one	C_10_H_16_O	1068	152.23	4.56 ± 0.30 ^b^	6.26 ± 0.37 ^a^	2.65 ± 0.43 ^c^
	(.+/−.)-Artemisia alcohol	3,3,6-Trimethyl-1,5-heptadien-4-ol	C_10_H_18_O	1090	154.24	5.02 ± 0.33 ^ab^	5.51 ± 0.37 ^a^	4.61 ± 0.02 ^b^
Total OD						9.58	11.77	7.26
Total						75.81	74.77	75.86

The values presented are means ± SD derived from three independent biological replicates. Letters following the content values of the same compound in different treatments of *A. argyi* leaves denote significant differences at *p* = 0.05. “Control leaves” denotes freshly dried *A. argyi* leaves. The essential oil yields were 7.40 ± 0.99 mg/g·DW for fermented leaves, 4.70 ± 0.71 mg/g·DW for control leaves, and 12.50 ± 0.77 mg/g·DW for aged leaves. While GC analysis identifies the primary components in the essential oils, it does not detect nonvolatile compounds or macromolecular substances such as sugars, proteins, and certain partially volatile esters. Therefore, the GC analysis only captures a significant portion of the overall composition. The retention index used for identifying target compounds was the Kovats retention index. MT, OMT, ST, OST, AC, and OD stand for monoterpene, oxygenated monoterpene, sesquiterpene, oxygenated sesquiterpene, aromatic compound, and olefin and derivative, respectively.

## Data Availability

The original contributions presented in this study are included in the article. Further inquiries can be directed to the corresponding author.

## Abbreviations

The following abbreviations are used in this manuscript:

SSF	Solid-state fermentation
LSF	Liquid-state fermentation
GC-MS	Gas Chromatography-Mass Spectrometry
IC50	Half maximal inhibitory concentration
DW	Dry weight
MT	Monoterpene
OMT	Oxygenated monoterpene
ST	Sesquiterpene
OST	Oxygenated sesquiterpene
AC	Aromatic compound
OD	Olefin and derivative
